# Author response to: Comment on: Cost–utility analysis of normothermic and hypothermic *ex-situ* machine perfusion in liver transplantation

**DOI:** 10.1093/bjs/znac261

**Published:** 2022-10-14

**Authors:** Julia Zimmermann, Alexander W Carter

**Affiliations:** Department of Health Policy, London School of Economics and Political Science, London, UK; London School of Hygiene and Tropical Medicine, London, UK; Cambridge Transplant Unit, Cambridge University Hospitals NHS Foundation Trust, Cambridge, UK; Department of Health Policy, London School of Economics and Political Science, London, UK

Thank you for the response to our paper^1^ on the cost–utility of normothermic and hypothermic *ex-situ* machine perfusion systems in liver transplantation. We found that, at a willingness-to-pay (WTP) threshold of £20 000 per quality-adjusted life-year, current practice in England dominates the Metra™ (OrganOX, Oxford, UK) and Liver Assist™ (Organ Assist, Groningen, the Netherlands) perfusion systems^1^. The finding that there is a 65 per cent probability of current practice being more cost-effective than alternative perfusion systems at the WTP threshold is equivalent to saying that the likelihood of this finding being robust is better than the probability of a fair coin toss, that is 50 per cent. Uncertainty without a model is higher, and could be anywhere between 0 and 100 per cent.

The correspondence helpfully explores some areas of uncertainty that health economists typically classify as methodological and structural uncertainty. Structural uncertainty arises from the choice of analytic model and the model’s assumptions. We detailed structural assumptions and their rationale, such as the postoperative complications we considered, in the supplementary material^1^. Uncertainty arising from the chosen approach is understood as methodological uncertainty. We explored this type of uncertainty in the one-way sensitivity analysis^1^. This shows that varying methodological assumptions, such as the discount rate, time horizon or long-term outcome data source, does not change decision outcomes.

It is important to note that the results of economic evaluations are driven by cost estimates, which form the model inputs and are subject to parameter uncertainty. The costs themselves are driven by the wider market and regulatory systems relevant to the decision context/country for which a model is produced. We are pleased that our paper is facilitating a discussion around the drivers of cost(-effectiveness) in the changing technology landscape for liver transplantation. It is entirely feasible that new perfusion systems will be cheaper in future, warranting re-estimation of the base case that we modelled.

## Disclosure

The authors declare no conflict of interest.
